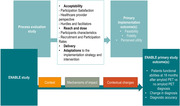# Enhancing the insights of the Patient‐and care‐related benefits of amyloid PET imaging ‐ ENABLE ‐ study through a process evaluation: a study protocol

**DOI:** 10.1002/alz.087334

**Published:** 2025-01-09

**Authors:** Alice Grazia, Doreen Goerss, Annika Spottke, Henning Boecker, Marcel Daamen, Erika Graf, Jörg Sahlmann, Ralph Buchert, Wolfgang Mohnike, Konrad Mohnike, Frank Jessen, Bernd J Krause, Marina Boccardi, Stefan Teipel, Jens Kurth

**Affiliations:** ^1^ Deutsches Zentrum für Neurodegenerative Erkrankungen (DZNE), Rostock‐Greifswald Standort, Rostock Germany; ^2^ Rostock University Medical Centre, Rostock Germany; ^3^ German Center for Neurodegenerative Diseases (DZNE), Rostock Germany; ^4^ Department of Psychosomatic Medicine, Rostock University Medical Center, Rostock Germany; ^5^ Deutsches Zentrum für Neurodegenerative Erkrankungen e. V. (DZNE) Bonn, Bonn Germany; ^6^ University of Bonn Medical Center, Bonn Germany; ^7^ German Center for Neurodegenerative Diseases (DZNE), Bonn Germany; ^8^ Institute of Medical Biometry and Statistics (IMBI), Faculty of Medicine – University Medical Center Freiburg, Freiburg Germany; ^9^ Department of Diagnostic and Interventional Radiology and Nuclear Medicine, University Medical Center Hamburg‐Eppendorf, Hamburg Germany; ^10^ University Hospital Hamburg‐Eppendorf, Hamburg Germany; ^11^ Diagnostic Therapeutic Center Berlin‐Frankfurter Tor, Berlin Germany; ^12^ Department of Psychiatry, University of Cologne, Medical Faculty, Cologne Germany; ^13^ Clinic for Nuclear Medicine, University Medicine Rostock, Rostock Germany; ^14^ Deutsches Zentrum für Neurodegenerative Erkrankungen e. V. (DZNE) Rostock/Greifswald, Rostock Germany; ^15^ Klinik und Poliklinik für Psychiatrie und Psychotherapie, University of Rostock, Rostock Germany; ^16^ Deutsches Zentrum für Neurodegenerative Erkrankungen e. V. (DZNE), site Rostock / Greifswald, Rostock Germany; ^17^ Department of Psychosomatic Medicine, University of Rostock, Rostock Germany; ^18^ Rostock University Medical Center, Rostock Germany

## Abstract

**Background:**

Amyloid PET imaging is an established diagnostic tool for Alzheimer's disease, but its successful integration into clinical practice requires a comprehensive understanding of its impact on patients and the healthcare system. In 2022, the coverage with evidence development (CED) ENABLE study has been approved by the German Federal Joint Committee (trial registration: DRKS00030839). The study is scheduled to start in early 2024. Primary outcome of ENABLE is the change in patients' functional abilities at 18 months after diagnosis with amyloid PET versus without amyloid‐PET. Here we describe the design of an ENABLE sub‐study aimed at performing a process evaluation.

**Method:**

We will conduct an outcome and process evaluation using a pre‐post descriptive design nested in ENABLE (Figure), based on the Medical Research Council's Process Evaluation (PE) framework. The PE will focus on acceptability and reach of the target groups of patients and care providers, as well as fidelity of delivery (% of adherence to protocol, study duration), contextual changes and mechanisms (e.g., ability to mirror routine care). We will collect patients’ data independently, but in parallel with ENABLE, using interviews, surveys and checklists to assess acceptance of the intervention, fidelity of adherence to the follow‐up procedures, perceived benefits and challenges. Healthcare providers and administrators will be interviewed to identify implementation hurdles and facilitators, contextual factors influencing uptake and underlying mechanisms. For data analysis we will employ a mixed‐methods approach.

**Result:**

Expected outcomes will include acceptability and adherence, barriers and facilitators to implementation, contextual factors influencing implementation, perceived utility of the intervention by patients and providers, and mechanisms by which the intervention can be delivered as part of normal care.

**Conclusion:**

This process evaluation can inform researchers on how to design future CED studies to be implemented into clinical care. The lessons learned during the development and approval process of the ENABLE project in Germany, as well as its process evaluation, can benefit other European CED studies and policy‐makers stakeholders in making informed decisions. Likewise, these findings can be generalized beyond the context of amyloid PET to other diagnostic biomarkers, ultimately improving the diagnosis and management of Alzheimer's disease.